# ID 160 - Alteração do Formato das Diretrizes Clínicas de Adenocarcinoma de Cólon e Reto no SUS

**DOI:** 10.5327/2237-9622.2025.v34s1.160

**Published:** 2025-11-25

**Authors:** Gláucia Teles de Araújo Bueno, Ludmila Andrade Alves Ferreira, Nicole Freitas de Mello, Marta da Cunha Souto Lobo Maior

**Keywords:** guia de prática clínica, protocolos clínicos, Sistema Único de Saúde, protocolos antineoplásicos, neoplasias de cólon, neoplasias retais

## Abstract

**Introdução:**

As Diretrizes Diagnósticas e Terapêuticas (DDT) e os Protocolos Clínicos e Diretrizes Terapêuticas (PCDT) em oncologia são documentos baseados em evidências, desenvolvidos pelo Ministério da Saúde, para orientar as melhores práticas clínicas no Sistema Único de Saúde (SUS). Devido ao sistema diferenciado de financiamento da oncologia, as DDT orientam práticas e evidências, conferindo maior autonomia de escolha aos serviços de saúde. Já os PCDT estabelecem critérios para o uso de procedimentos e tecnologias financiadas pelo SUS, reduzindo as iniquidades e harmonizando condutas. Este trabalho tem como objetivo analisar o processo de transição da diretriz clínica de adenocarcinoma de cólon e reto de DDT para PCDT.

**Método:**

Descrição do processo de desenvolvimento do PCDT de adenocarcinoma de cólon e reto a partir das DDT. Foram consultadas orientações metodológicas do Ministério da Saúde para a elaboração e atualização de diretrizes clínicas, a DDT vigente para a neoplasia e diretrizes clínicas nacionais e internacionais publicadas pelas seguintes instituições: (NCCN) 2024, (ESMO) 2022, Sociedade Brasileira de Oncologia Clínica (Sboc) 2024. Também foram consultados os protocolos institucionais do Instituto Nacional de Câncer (Inca) 2022 e do Instituto do Câncer de São Paulo (Icesp) 2019, além de um painel de especialistas.

**Resultados:**

A partir das recomendações das DDT vigentes e das diretrizes clínicas e protocolos institucionais selecionados, foram estabelecidos critérios para o cuidado dos usuários com adenocarcinoma de cólon e reto, baseados nas melhores evidências disponíveis. As recomendações foram adequadas ao contexto do SUS, considerando as decisões de incorporação ou não incorporação de tecnologias pelo Ministério da Saúde, e apresentadas ao painel de especialistas. Assim, foi possível alinhar as recomendações da proposta de PCDT e validar os critérios estabelecidos.

**Conclusão:**

A alteração do formato do documento – da DDT do câncer de cólon e reto para o PCDT de adenocarcinoma de cólon e reto – é permeada por desafios. Uma das principais limitações observadas foi a alta variabilidade das alternativas terapêuticas disponíveis nas diretrizes internacionais e nacionais. Além disso, essas diretrizes foram elaboradas em contextos de serviços específicos, de outros sistemas de saúde ou sem um processo de avaliação de tecnologias pelo Ministério da Saúde. Assim, os contextos distintos aos do PCDT dificultaram a comparação das orientações. Em contrapartida, a análise comparativa com as demais diretrizes foi um importante facilitador do processo de transição de formato da diretriz para o PCDT. A partir da discussão com o painel de especialistas, foi possível desenvolver critérios e recomendações robustas para o cuidado e adequadas ao contexto do SUS. Identificar, avaliar e priorizar tecnologias para o tratamento do câncer no Brasil é essencial para garantir, de forma equânime e sustentável, o acesso às melhores práticas em saúde baseadas em evidências. Com o novo PCDT, espera-se que gestores e profissionais de saúde tenham mais subsídios sobre as condutas mais adequadas no diagnóstico e no tratamento do adenocarcinoma de cólon e reto.

